# A Computational Model of Interactions Between Neuronal and Astrocytic Networks: The Role of Astrocytes in the Stability of the Neuronal Firing Rate

**DOI:** 10.3389/fncom.2019.00092

**Published:** 2020-01-22

**Authors:** Kerstin Lenk, Eero Satuvuori, Jules Lallouette, Antonio Ladrón-de-Guevara, Hugues Berry, Jari A. K. Hyttinen

**Affiliations:** ^1^BioMediTech, Faculty of Medicine and Health Technology, Tampere University, Tampere, Finland; ^2^Institute for Complex Systems (ISC), National Research Council (CNR), Sesto Fiorentino, Italy; ^3^Department of Physics and Astronomy, University of Florence, Sesto Fiorentino, Italy; ^4^Department of Human Movement Sciences, MOVE Research Institute Amsterdam, Vrije Universiteit Amsterdam, Amsterdam, Netherlands; ^5^INRIA, Villeurbanne, France; ^6^LIRIS UMR5205, University of Lyon, Villeurbanne, France

**Keywords:** simulation, neuron, astrocyte, network, calcium signaling, gliotransmission

## Abstract

Recent research in neuroscience indicates the importance of tripartite synapses and gliotransmission mediated by astrocytes in neuronal system modulation. Although the astrocyte and neuronal network functions are interrelated, they are fundamentally different in their signaling patterns and, possibly, the time scales at which they operate. However, the exact nature of gliotransmission and the effect of the tripartite synapse function at the network level are currently elusive. In this paper, we propose a computational model of interactions between an astrocyte network and a neuron network, starting from tripartite synapses and spanning to a joint network level. Our model focuses on a two-dimensional setup emulating a mixed *in vitro* neuron-astrocyte cell culture. The model depicts astrocyte-released gliotransmitters exerting opposing effects on the neurons: increasing the release probability of the presynaptic neuron while hyperpolarizing the post-synaptic one at a longer time scale. We simulated the joint networks with various levels of astrocyte contributions and neuronal activity levels. Our results indicate that astrocytes prolong the burst duration of neurons, while restricting hyperactivity. Thus, in our model, the effect of astrocytes is homeostatic; the firing rate of the network stabilizes to an intermediate level independently of neuronal base activity. Our computational model highlights the plausible roles of astrocytes in interconnected astrocytic and neuronal networks. Our simulations support recent findings in neurons and astrocytes *in vivo* and *in vitro* suggesting that astrocytic networks provide a modulatory role in the bursting of the neuronal network.

## Introduction

Neuroscience research has focused for long on neurons and their interacting networks. However, the brain also consists of a large number of other different cell types, among which glial cells represent roughly 50% of the brain cells (Kettenmann and Verkhratsky, 2008; Azevedo et al., 2009). Among glial cells, astrocytes offer metabolic support to neurons, regulate the extracellular ions like potassium and calcium released upon neuronal activity (Dallérac et al., 2013; Hertz et al., 2015) and uptake neurotransmitters (Bezzi et al., 1998; Araque et al., 2001; Perea and Araque, 2007; Volterra et al., 2014). Indeed, some of the synapses of the central nervous system are contacted by astrocytes that wrap around them, thus forming a structural ensemble called the tripartite synapse: presynaptic neuron, post-synaptic neuron and the ensheathing astrocyte (Araque et al., 1999).

Intracellular calcium (Ca^2+^) transients are a prominent readout signal of astrocyte activity, and happens at different time scales (Kastanenka et al., 2019). They may be triggered by neuronal activity (Di Castro et al., 2011; Dallérac et al., 2013). At glutamatergic synapses, inositol 1,4,5-trisphosphate (IP_3_) is released in the astrocyte cytoplasm after some of the presynaptically released glutamate binds to metabotropic glutamate receptors in the astrocytic plasma membrane. The released IP_3_ binds to IP_3_- and Ca^2+^-gated Ca^2+^ channels in the membrane of the endoplasmic reticulum, thus leading to a Ca^2+^ elevation in the astrocyte cytosol. In return, these transient changes in the level of free cytoplasmic Ca^2+^ lead to the opening of further IP_3_ channels in a Ca^2+^-induced Ca^2+^ release (CICR) mechanism that further amplifies Ca^2+^ release from the endoplasmic reticulum. The internal calcium pathways may also be linked to the release by the astrocyte of so-called gliotransmitters—like glutamate, D-serine, adenosine triphosphate (ATP), and GABA (γ-aminobutyric acid)—that influence the activity of the contacted neurons (Pasti et al., 2001; Henneberger et al., 2010; Zorec et al., 2012; Araque et al., 2014; Sahlender et al., 2014).

Neuron-astrocyte interactions are thought to occur—or be initiated—at the thinnest astrocytic processes/branchlets (Bazargani and Attwell, 2016; Bindocci et al., 2017). Furthermore, astrocytes themselves form interconnected networks via gap junctions. Gap junctions formed by connexins build a pore through the cell membranes of two adjacent astrocytes, joining their cytosols and letting through certain sized molecules, including IP_3_ and potassium ions (Fellin, 2009; Giaume et al., 2010). The modulating effect of astrocytes on neuronal network activity has been shown in several *in vitro* experiments. Tukker et al. (2018) showed that the spike and burst rates were reduced in matured networks with glutamatergic neurons and astrocytes compared to glutamatergic neurons only. Co-cultured human stem cell-derived neurons and astrocytes exhibited a marginal decrease in the spike rate and an increase in the burst rate and duration, while the number of spikes per bursts was constant when more astrocyte were present in the network (Paavilainen et al., 2018).

Dedicated computational models of the cross-talk between neuron networks and astrocytes have been successfully employed to explore specific issues related to neuron-astrocyte interactions (for a review, see Oschmann et al., 2018). For example, Amiri et al. (2013) combined two coupled Morris-Lecar neuron models and the dynamic astrocyte model of Postnov et al. (2009). They simulated 50 pyramidal neurons, 50 interneurons, and 50 astrocytes, connected in a chain-like manner, with each astrocyte connected to one pyramidal cell, one interneuron, and one neighboring astrocyte via gap junctions. This study suggested that increasing the influence of the astrocytes toward the neurons leads to a reduction of the synchronized neuronal oscillations. Valenza et al. (2013) developed a transistor-like description of the tripartite synapse and also included short-term synaptic plasticity for excitatory synapses. They simulated a network containing 1,000 neurons and 1,500 astrocytes where at least one astrocyte was linked to each neuron. This model was able to produce spontaneous polychronous activity—i.e., reproducible time-locked but not synchronous firing—in neural groups.

More recently, Aleksin et al. (2017) presented neural network simulation software called ARACHNE, which is partially based on the NEURON environment. This model includes a chain-like structure in ring form, basic equations for the internal astrocytic dynamics and extracellular diffusion of gliotransmitters (volume transmission). Additionally, Stimberg et al. (2019) recently presented how the Brian 2 simulator can be used to model networks of interacting neurons and astrocytes. The authors notably showed how, after a period of high external stimulation of the neurons, gliotransmission can maintain a high level of neuronal activity and firing synchrony for several seconds after the end of the external stimulation. Although those modeling studies clearly advanced our understanding of the interaction between neuron networks and astrocyte networks, few of them included all three of the following significant ingredients of astrocyte networks: (i) Astrocytes form gap junction-based networks that convey calcium-based signals as waves (Charles et al., 1996; Fellin, 2009); (ii) each astrocyte contacts a large number of synapses, estimated to be up to 100,000 synapses per astrocyte in rat hippocampus (Bushong et al., 2002); and (iii) astrocytes can release distinct types of gliotransmitters (Di Castro et al., 2011; Sahlender et al., 2014; Schwarz et al., 2017), for instance, a single hippocampal astrocyte can co-release both excitatory (glutamate) and depressing gliotransmitters (adenosine), thus exerting a biphasic control of the synapse (Covelo and Araque, 2018).

In this work, we develop a mathematical model of combined astrocyte-neuron networks to study the role of astrocyte networks on the modulation of the neuronal firing rate. In our model, which we call INEXA, astrocytes regulate neuronal communication through the tripartite synaptic function, and they can release both excitatory and depressing gliotransmitters in response to synaptic activity. We moreover introduce the biological property that each astrocyte is connected to hundreds of synapses. In a two-dimensional spatial setup emulating neuron-astrocyte co-cultures, we study how astrocytes control the homeostasis in neuronal networks by increasing the ratio of astrocytes. Further, we assess how the level of neuronal input can alter both the neuronal firing rate and the astrocytic calcium activity.

## Methods

**Figure 1:**
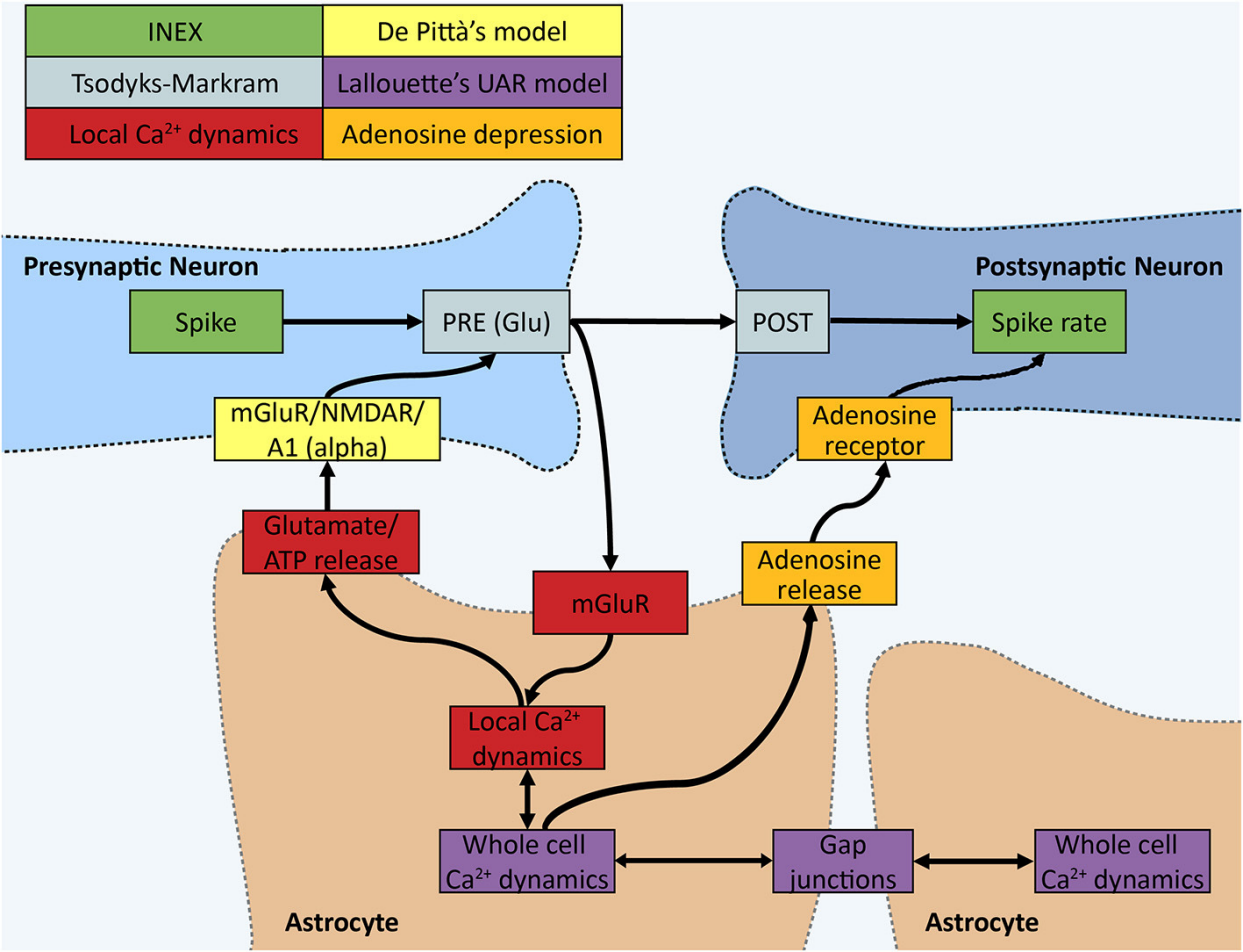
Schematic of the INEXA model. The colors represent different parts of the simulator. In the INEX model by Lenk et al. (green), the spike has an effect on the spiking rate of the post-synaptic neuron through the synaptic weight. We added the Tsodyks-Markram (gray) synapse model together with De Pittà's astrocyte gliotransmitter interface (yellow). To monitor the synapse activity, a local calcium dynamics simulator (red) was added to each synapse, which is controlled by an astrocyte. Local astrocyte dynamics control gliotransmission to the synapse. All the local calcium simulators can have an effect on the whole cell calcium signaling modeled in the UAR model (purple) by Lallouette et al. In the UAR model, the calcium activity can spread across cells, mimicking calcium wave propagation through gap junction-mediated IP_3_ diffusion. A whole cell calcium signal sets the local calcium dynamics to a high calcium state and ATP (quickly degraded into adenosine, orange part) is released into the extracellular space by the astrocyte to restrict the spiking of neurons nearby.

We developed a computational model that integrates the key components of astrocyte-neuron modulation (Figure 1). In section INEXA: A Computational Framework to Model Neuron-Astrocyte Networks, we describe the full INEXA model including the neuronal and astrocytic components and the manner in which they are coupled with each other. In section Numerical and Analysis Methods, we describe the numerical methods for analyzing the simulated neuronal and astrocytic activity. The outline of the simulations is specified at the end of section Numerical and Analysis Methods.

### INEXA: A Computational Framework to Model Neuron-Astrocyte Networks

#### Neuronal Components

##### Neuronal activity

Our goal was to develop a model of neuronal spiking in primary mixed cultures (i.e., containing neurons and astrocytes) grown on multielectrode arrays (MEAs). We based our model on the phenomenological INEX model (Lenk, 2011), since it was initially built for *in vitro* neuronal networks. INEX is a stochastic cellular automaton in which inhibitory and excitatory neurons are connected to each other via synapses. Moreover, noise is applied to each neuron to reproduce background activity. In this fashion, INEX is a computationally-light model that has also been shown of well-reproducing neuronal dynamics of neuronal cultures plated on MEAs (Lenk, 2011; Lenk et al., 2016). For all these reasons, we adopted it as a starting platform for neuronal networks to be complemented by astrocytic coupling.

Briefly, INEX is a discrete-time model with a time step *t*_*k*_ = Δ*t*. The instantaneous firing rate λ_*i*_ of neuron *i* in time slice *t*_*k*_ is calculated as (Lenk, 2011):

(1) $${\text{λ}}_{i}\left({\text{t}}_{k}\right)=\;\max\;\left(0,\;c_{i}+\sum_{j}y_{ij}s_{j}\left({\text{t}}_{k-1}\right)\right)$$

where *c*_*i*_ is the noise of neuron *i* and *y*_*ij*_ the synaptic strength from presynaptic neuron *j* to post-synaptic neuron *i*. For each neuron, the value of *c*_*i*_ was set independently by sampling from a triangular distribution between 0 and an upper bound, *C*_*max*_. The value of *C*_*max*_ depends on the simulation, in order to explore the effects of the noise level (see Table 1). The term *s*_*j*_ indicates whether a spike has been emitted by neuron *j* in the previous time step (*s*_*j*_ = 1 if a spike has been emitted, else *s*_*j*_ = 0).

**Table 1:** Basic simulation parameters.

**Parameter**	**Value**	**Unit**	**Definition**
*C*_**max**_	0.01; 0.02; 0.03	–	Upper boundaries for the three noise levels
$Y_{max}^{+}$	0.7	–	Upper boundary for excitatory synaptic weights
$Y_{max}^{-}$	−0.7	–	Upper boundary for inhibitory synaptic weights
Ω_*d*_	4.0405	*s*^−1^	Recovery rate of synaptic vesicles
Ω_*f*_	2.0	*s*^−1^	Rate of synaptic facilitation
α	0.7	–	Effect parameter of astrocyte regulation of synaptic release
Ω_*g*_	0.077	*s*^−1^	Recovery rate of gliotransmitter receptors
g_r_	0.3	–	Fraction of unbound receptors recruited by gliotransmission
Ca_th_	0.1	–	Calcium threshold for gliotransmitter release
Ω_*acc*_	0.05	–	Accumulation rate between IP_3_ and Ca^2+^
Ω_I_P__3__	152.3	*s*^−1^	IP_3_ degradation rate
Astrocytes	28; 63; 107	–	Number of astrocytes for NN+A(10%), NN+A(20%) and NN+A(30%), respectively
M	5	–	Multiplier between astrocyte near synapse and whole astrocyte self-induced IP_3_ flux
Connection distance	100	*μm*	Maximum distance between two connected astrocytes
τ_*A*_	1.5	*s*	Average activation time of an astrocyte
τ_*R*_	7.0	*s*	Average refractory time of an astrocyte
τ_*U*_	5.0	*s*	Average time needed to activate an astrocyte
b_0_	0.02	–	Slope of the activation threshold
b_1_	0.205	–	Intercept of the activation threshold
y_Astro_	0.01	–	Depressing signal applied by astrocytes
Culture area	[750 750 10]	*μm*	Resamples MEA electrode area for each dimension
Min. neuron distance	10	*μm*	Minimum distance between randomly placed neurons
Min. astrocyte distance	30	*μm*	Minimum distance between randomly placed astrocytes
σ_*N*_	200	*μm*	Standard deviation of neuronal connections
σ_*A*_	150	*μm*	Standard deviation of astrocyte-neuron connections without limiter
*d*_*A*_	70	*μm*	Limiter cutting the Gaussian standard deviation connection probability set by standard deviation
T	300	*s*	Simulation time

*To keep the model as computationally light as possible and to maintain biological plausibility, the previously introduced models are combined using relatively simple components that are not accurate descriptions of the processes, but rather descriptive. The parameters in INEX are phenomenological and were fixed using brute force to find sets of parameters that produced results in reasonable ranges (Lenk et al., 2016). By adding the Tsodyks-Markram presynapse model, we introduced short-term memory at the level of individual synapses. The parameters are adapted from the model of De Pittà et al. (2011), which uses approximations of the local astrocytic calcium and IP_3_. For the implementation of the UAR model, the parameters described in the supplementary part of the paper by Lallouette et al. (2014) are used. The values of the adenosine depression are chosen in such a way, that the astrocyte can reduce the probability of the neuronal spiking but cannot shut it down completely (Yoon and Lee, 2014). The basic principle of building our neuronal and astrocytic network topologies is that it reasonably represents a cultured network on an in vitro multielectrode array (Wallach et al., 2014; Paavilainen et al., 2018; Tukker et al., 2018). The figure of 250 neurons was found to be computationally fast enough, since several runs are needed to optimize parameters and produce comparable statistics. Astrocytes are set randomly but at least 30 μm apart. The simulation does not take into account the exact microdomains (Bushong et al., 2002; Agarwal et al., 2017) occupied by astrocytes, but assumes that the shape of the astrocytes allows them to occupy spaces that are non-uniformly spread around the cell soma*.

Note that, in our model, each excitatory presynapse is connected to an astrocyte with a probability that decreases with the distance between the synapse and the soma of the astrocyte (see Neuron and Astrocyte Network Spatial Topologies). We thus have thus adapted Equation (1) to account for the effect of astrocytes on the synapse (see Glial Components).

The probability *P*_*i*_(*t*_*k*_) for neuron *i* to emit a spike during time step *k*—i.e., between *t*_*k*_ and *t*_*k*_ + Δ*t*—is then modeled as an inhomogeneous Poisson process with rate λ_*i*_(*t*_*k*_):

(2) $$P_{i}\left({\text{t}}_{k}\;\right)=\;e^{-{\text{λ}}_{i}\left({\text{t}}_{k}\;\right)\Delta t}\cdot {\text{λ}}_{i}\left({\text{t}}_{k}\;\right)\Delta t.$$

Here, we used Δ*t* = 5 ms to cover the typical duration of an action potential and the subsequent refractory period. Thus, we neglected the probability that more than one spike may be emitted by a given neuron during a single time step. At the benefit of computational efficiency, a time step as large as Δ*t* = 5 ms can be adopted and the INEX network model can still reliably simulate neuronal activity recorded in MEA cultures (Lenk, 2011; Lenk et al., 2016).

##### Presynaptic dynamics

For the dynamics of presynaptic neuronal release, we used the Tsodyks-Markram (TM) presynapse model (Tsodyks et al., 1998). The TM model consists of two variables, *x* and *u*, describing the fraction of neurotransmitters available in the presynaptic terminal and the fraction of these available neurotransmitters that are ready for release (which can be seen as the release probability), respectively. We have discretized the original TM equations and thus, for each synapse *ij* applied:

(3) $$\begin{array}{l} x_{ij}\left({\text{t}}_{k}\right)=\left(x_{ij}\left({\text{t}}_{k-1}\right)-RR_{ij}\left({\text{t}}_{k}\right)\right)\\ \;+\left[1-\left(x_{ij}\left({\text{t}}_{k-1}\right)-RR_{ij}\left({\text{t}}_{k}\right)\right)\right]\left(1-e^{-\Omega_{d}\text{t}}\right), \end{array}$$

(4) $$u_{ij}\left({\text{t}}_{k}\right)=\left[\left(1-u_{ij}\left({\text{t}}_{k-1}\right)\right)U_{ij}^{*}\left({\text{t}}_{k}\right)s_{j}\left({\text{t}}_{k}\right)+u_{ij}\left({\text{t}}_{k-1}\right)\right]e^{-\Omega_{f}\Delta t},$$

(5) $$\begin{array}{l} RR_{ij}\left(t_{k}\right)=x_{ij}\left(t_{k-1}\right)[\left(1-u_{ij}\left({\text{t}}_{k-1}\right)\right)U_{ij}^{*}(t_{k})s_{j}\left(t_{k}\right)\\ \;+u_{ij}\left(t_{k-1}\right)]s_{j}\left(t_{k}\right), \end{array}$$

where Ω_*d*_ represents the rate of reintegration of neurotransmitters in the presynaptic terminal, Ω_*f*_ the rate of decrease of release probability, *RR*_*ij*_ the fraction of released neurotransmitters, and $U_{ij}^{*}$ denotes the maximal increment of the ready-for-release fraction triggered by the arrival of a presynaptic spike.

The discretization of the TM equations was achieved by assuming that neuronal spikes happen at the very start of the 5 ms time steps. Just after a spike at the start of time step *t*_*k*_, the release probability *u* takes the value $\left(1-u_{ij}\left(t_{k-1}\right)\right)U_{ij}^{*}{\left(t_{k}\right)s}_{j}\left(t_{k}\right)+u_{ij}\left(t_{k-1}\right)$: the sum of its previous values at the end of time slice *t*_*k*−1_ and the additional recruitment of a fraction $U_{ij}^{*}$ of the previously non-recruited available resources. This temporary value *u* just after a spike is used to compute: (1) the value of *u* at the end of the time step *t*_*k*_ (Equation 4) by applying a simple exponential decay term, and (2) the released resources for this time slice (Equation 5) by simply multiplying it by the fraction of available resources *x* at the end of time step *t*_*k*−1_. The available resources at the end of time step *t*_*k*_ are then computed (Equation 3) by subtracting the released resources from the available resources at the end of time step *t*_*k*−1_ and then applying an exponential term accounting for the reintegration of resources. In our model, the value of $U_{ij}^{*}$ in turn varies with time depending on gliotransmitter release by the astrocyte that enwraps the synapse (see Glial Components).

The strength of the synapse *y*_*ij*_ was chosen to be directly proportional to the fraction of released resources *RR*_*ij*_:

(6) $$y_{ij}\left(t_{k}\right)\;=\;Y_{\max}\cdot RR_{ij}\left(t_{k}\right),$$

where *Y*_*max*_ represents the largest value that the inhibitory ($Y_{max}^{-}$) or excitatory ($Y_{max}^{+}$) strength of a synapse can take.

#### Glial Components

##### Regulation of synaptic dynamics by gliotransmission

The questions of whether gliotransmitters are actually released by astrocytes and whether released gliotransmitters do contribute to the modulation of neuronal activity are still debated (see e.g., the two main perspectives expressed in Fiacco and McCarthy, 2018; Savtchouk and Volterra, 2018). In particular, the mechanisms by which gliotransmitters can be released are unclear, although both calcium-dependent vesicular release and channel-based release have been evidenced (Sahlender et al., 2014). However, an increasing number of experiments confirm that astrocytes are not just passive read-out units; they are heavily involved in the modulation of neuronal synapses and their activity (Fellin et al., 2004; Perea et al., 2009; Clarke and Barres, 2013). These results show that depending on the type of receptors expressed by the presynaptic and post-synaptic neurons, astrocyte-released glutamate can either potentiate (via presynaptic or extrasynaptic NMDAR) or depress the synapse (via presynaptic mGluR; Jourdain et al., 2007; Fellin, 2009; Bonansco et al., 2011; Min et al., 2012; Papouin and Oliet, 2014).

In addition to glutamate, astrocytes can also release purines such as ATP and adenosine (Newman, 2003; Bowser and Khakh, 2007; Lorincz et al., 2009; Hines and Haydon, 2014). Moreover, extracellular ATP of astrocytic origin could also be hydrolyzed into adenosine. By binding to A1 receptors on the presynaptic terminal, adenosine has been shown to reduce synaptic strength (Boddum et al., 2016; Savtchouk and Volterra, 2018). In a very similar way, astrocytes have also been reported to release GABA, a phenomenon involved in tonic inhibition (McIver et al., 2013), probably via calcium-regulated channels (Lee et al., 2010). Therefore, converging experimental evidence suggests that astrocytes release gliotransmitters that can either increase or decrease synaptic activity. In neurons, segregation between inhibitory and excitatory transmission is the rule. Excitatory neurons usually release glutamate, whereas inhibitory neurons release GABA, although exceptions exist, including the co-release of GABA and glutamate by the same presynaptic synapse (Shrivastava et al., 2011). However, the only available related experimental report on astrocytes concluded against segregation: in hippocampal slices, it was shown that a single astrocyte can release both glutamate and adenosine, thus mediating an initial potentiation of the synapse, followed by longer-lasting depression (Covelo and Araque, 2018). Lorincz et al. (2009) and Newman (2003) suggested in their studies that adenosine could also bind to A1 receptors post-synaptically and trigger neuronal inhibition through G protein-coupled inwardly rectifying K^+^ channels.

In the present work, we explore the effects of such a non-segregated gliotransmitter release, assuming that a single astrocyte can release both potentiating and depressing gliotransmitters. Therefore, we assumed that gliotransmitter release is not segregated in astrocytes—i.e., a single astrocyte can release both potentiating and depressing gliotransmitters at the same synapse. To model the effect of depressing gliotransmitters, we added to each excitatory synapse contacted by an astrocyte an additional depressing signal from the astrocyte that could be mediated by adenosine (Newman, 2003; Lorincz et al., 2009). This was accounted for in the model by a term modulating the synaptic weights *y*_*Astro*_, that modified Equation (1) to:

(7) $${\text{λ}}_{i}\left(t_{k}\right)=\text{ max}\left(0,\;c_{i}+\sum_{j}y_{ij}\cdot s_{j}\left(t_{k-1}\right)-\sum_{j}y_{Astro}\cdot A_{ija}\left(t_{k-1}\right)\right),$$

where *A*_*ija*_ = 1 if synapse *ij* is enwrapped by astrocyte “*a*” and if astrocyte “*a*” was in the active state at the previous time-step, else *A*_*ija*_ = 0 (the conditions for astrocyte activation are detailed in section Astrocytic network dynamics). Therefore, if an astrocyte is close enough to synapse *ij* to enwrap it, the astrocyte exerts a depressing effect, *y*_*Astro*_, on the synapse as long as the astrocyte is in the active state. Note that the duration of the resulting depression is set by the time spent by the astrocyte in the active state. In our simulations, this activation time is usually large (seconds, **Figure 5D**).

To model the effects of potentiating gliotransmitter release on the presynaptic part, we followed a paper by De Pittà et al. (2011), wherein a single parameter, α, is used to describe the effects of the co-operation of multiple receptors. We considered that ATP and glutamate are released in a single release event and that their binding kinetics to their receptors are fairly similar. As in De Pittà et al. (2011) and De Pittà (2019), α modifies the value of $U_{ij}^{*}\left(t_{k}\right)$, which describes the effect of gliotransmission on the synaptic release probability (see section “Presynaptic dynamics”):

(8) $$U_{ij}^{*}\left(t_{k}\right)\;=\;\frac{{y_{ij}}_{base}}{Y_{\text{max}}}\;\cdot \;\left(1-g_{ij}\left(t_{k}\right)\right)+\alpha \cdot g_{ij}\left(t_{k}\right),$$

where *g*_*ij*_(*t*_*k*_) is the fraction of bound presynaptic gliotransmitter receptors (see section Astrocyte response to presynaptic stimulations). In the absence of gliotransmission, i.e., for the synapses that are not connected by an astrocyte, *g*_*ij*_*(t*_*k*_*)* = 0 for all time steps *t*_*k*_, so that $U_{ij}^{*}$ is set to a constant value ($U_{ij}^{*}=\;\frac{{y_{ij}}_{base}}{Y_{max}}$). The value of α sets the influence of gliotransmission on presynaptic release: depending on its value, α can account for depressing gliotransmission ($0<\alpha <\frac{{y_{ij}}_{base}}{Y_{max}}$) or potentiating gliotransmission ($\frac{{y_{ij}}_{base}}{Y_{max}}<\alpha <$1). Here, our focus is on the non-segregated gliotransmitter release as reported by Covelo and Araque (2018), where a single astrocyte can sequentially elicit sequentially a potentiation of the synaptic weights followed by a longer-lasting depression. The latter phase is accounted for by the term *y*_Astro_ *A*_*ija*_ in Equation (7). We thus emulate the initial potentiation phase by setting α to a potentiating value (α = 0.7 while $\frac{{y_{ij}}_{base}}{Y_{max}}<0.7;$ see below and Table 1). The parameter *y*_*ij*_*base*__ is the basal synaptic strength of synapse *ij* in the absence of gliotransmission: a spike arriving at the presynaptic terminal of synapse without an adjacent astrocyte that has fully recovered from its previous activity (i.e., *x*_*ij*_(*t*_*k*−1_) = 1 and *u*_*ij*_(*t*_*k*−1_) = 0), yields *y*_*ij*_(*t*_*k*_) = *y*_*ij*_*base*__ from Equations (5–7) above. In our model, *y*_*ij*_*base*__ was sampled randomly from a triangular distribution (0 ≤ *y*_*ij*_*base*__ ≤ 0.7). The triangular distribution was a simplification of the Gaussian distribution, which guaranteed the positivity of the values.

##### Astrocyte response to presynaptic stimulations

Calcium transients in astrocytes can be classified into at least two main types. Transient calcium elevations can happen independently of neuronal activity (spontaneous transients) or they can be triggered by the activity of nearby presynaptic neurons (activity-driven transients) (Perea et al., 2009; Wallach et al., 2014). Although astrocytic calcium signals can invade the whole cell (Volterra et al., 2014; Bindocci et al., 2017) and even be transmitted to coupled astrocytes (Parri et al., 2001), some calcium signals are restricted to the neighborhood of their origin. Thus, they cause calcium elevation locally, at a range of only one or a few synapses (Perea et al., 2009; Di Castro et al., 2011; Bindocci et al., 2017).

To account for the response of the astrocyte to glutamate release by the presynaptic element of the tripartite synapse, we modeled each astrocyte as a multi-compartment cell with local areas and a soma. Local area *ija* of astrocyte “*a*” represents the subpart of the astrocyte that is in direct contact with synapse *ij* and is associated to its own local IP_3_ and calcium dynamics. Here, we expressed those local IP_3_ and calcium transients using a simplified version of the astrocyte IP_3_/calcium dynamics described by De Pittà and co-workers (De Pittà et al., 2008, 2019). The variables [IP_3_] and [Ca^2+^] denote the concentrations of IP_3_ and Ca^2+^, respectively in local area *ija* of astrocyte “*a*”. Upon emission of a presynaptic spike by neuron *j*, [*IP*_3_]_*ija*_(*t*_*k*_) is incremented by a value that depends on the amount of resources released into the synaptic cleft, *RR*_*ija*_(*t*_*k*_). [*IP*_3_]_*ija*_(*t*_*k*_) then decreases exponentially fast at rate Ω_*IP*_3__:

(9) $$\begin{array}{l} {\left[IP_{3}\right]}_{ija}\left(t_{k}\right)=\;{\left[IP_{3}\right]}_{ija}\left(t_{k-1}\right)\;\cdot e^{-\Omega_{{\text{IP}}_{\text{3}}}\;\Delta t}\\ \;+\left(1-{\left[IP_{3}\right]}_{ija}\left(t_{k-1}\right)\;\cdot e^{-\Omega_{{\text{IP}}_{\text{3}}}\;\Delta t}\right)\;\cdot RR_{ij}(t_{k}). \end{array}$$

To express the local calcium dynamics, we simplified the dynamics further and chose to focus on amplitude-modulated (AM) astrocyte responses to stimulation (De Pittà et al., 2008). Thus, larger IP_3_ concentrations translate into larger calcium concentrations and not larger oscillation frequencies (De Pittà et al., 2008). To account for the expected slow time scale of the calcium-release machinery (up to seconds), we made the local calcium dynamics ${\left[Ca^{2+}\right]}_{ija}\left(t_{k}\right)$ converge to [*IP*_3_]_*ija*_(*t*_*k*_) with time scale Ω_*acc*_:

(10) $$\begin{array}{l} {\left[Ca^{2+}\right]}_{ija}(t_{k})={\left[Ca^{2+}\right]}_{ija}(t_{k-1})+\Omega_{acc}\cdot ({\left[IP_{3}\right]}_{ija}(t_{k})\\ \;-{\left[Ca^{2+}\right]}_{ija}(t_{k-1})). \end{array}$$

Gliotransmission occurs when the local calcium concentration exceeds the threshold ${\left[Ca^{2\;+}\right]}_{th}$:

(11) $$g_{ij}\left(t_{k}\right)=\{\begin{array}{c} \left(g_{ij}\left(t_{k-\;1}\right)+\left(1-g_{ij}\left(t_{k-1}\right)\right)\;\cdot g_{r}\right)\;\cdot e^{-\Omega_{g}\Delta t}\text{ if }{\left[Ca^{2+}\right]}_{ija}\left(t_{k-1}\right)\;<{\left[Ca^{2+}\right]}_{th}<{\left[Ca^{2+}\right]}_{ija}\left(t_{k}\right)\\ \;g_{ij}\left(t_{k-1}\right)\;\cdot e^{-\Omega_{g}\Delta t}\text{ otherwise} \end{array}\;,$$

where the condition for ${\left[Ca^{2+}\right]}_{ija}$ ensures the absence of a new gliotransmission event when calcium drops back below the threshold. In this equation, *g*_*ij*_(*t*_*k*_) is the fraction of bound presynaptic gliotransmitter receptors, *g*_*r*_ the fraction of unbound receptors recruited, and Ω_*g*_ the recovery rate of gliotransmitter receptors. For simplicity, and unlike in De Pittà et al. (2008), we consider a constant gliotransmission recruiting fraction.

##### Astrocytic network dynamics

To model astrocyte-astrocyte calcium signaling, we used the UAR model introduced by Lallouette et al. (2014, 2019). In the network model, each astrocyte is a node, and gap junctions are links between the nodes. In the UAR model, an astrocyte “*a*” can have three possible states *S*_*a*_: active state (A), inactive dormant state (U), and refractory (R), during which the cell cannot transmit calcium signals. At any time, the cell will be in one of these states. Transitions between states are probabilistic and depend on the propagation efficiency of coupled astrocytes. The propagation efficiency of an active astrocyte “*a*” is (Lallouette et al., 2014, 2019):

(12) $$\beta_{a}\left(t_{k}\right)=\;\{\begin{array}{c} \frac{1}{I_{a}(t_{k})}\text{ if }S_{a}\left(t_{k}\right)=A\\ 0\text{ else} \end{array}\;,$$

where *I*_*a*_(*t*_*k*_) is the number of astrocytes that are gap junction-coupled to “*a*” and are not in the active state *A*. The activation propensity of “*a*” is then obtained with:

(13) $$\gamma_{a}\left(t_{k}\right)\;=\;\theta_{a}\sum_{b\in \;N(\;a)}\beta_{b}\left(t_{k}\right)+\;\frac{\sum {\left[Ca^{2+}\right]}_{ija}}{N}\cdot M,$$

where N(a) is the set of astrocytes that are gap-junction-coupled to “*a*” and θ_*a*_ is the astrocyte activation threshold. The sum in the second term of the right-hand side of Equation (13) runs over all local areas *ija* composing astrocyte “*a*,” thus effectively adding up the calcium ${\left[Ca^{2+}\right]}_{ija}$ of each of the astrocyte's regions. These local responses are averaged over the whole astrocyte (*N* is the number of excitatory connections to astrocyte “*a*”) and scaled by a factor *M* to arrive at their contribution to the activation propensity. If the activation propensity of an astrocyte is larger than the threshold θ_*a*_, this astrocyte can activate. Following Lallouette et al. (2014), this threshold changes with the number of astrocyte neighbors *n*_*a*_ as:

(14) $$\theta_{a}(n_{a})\;=\;b_{0}n_{a}+b_{1},$$

where *b*_0_ denotes the slope of the activation threshold and *b*_1_ as the intercept of the activation threshold. The probability for astrocyte “*a*” to become active (U → A) at time step *t*_*k*_ is finally calculated as:

(15) $$P{\left(U\to A\right)}_{a}\left(t_{k}\right)\;=\;\{\begin{array}{c} \frac{\Delta t}{\tau_{A}}if\;{\text{γ}}_{a}\left(t_{k}\right)>\;\theta_{a}\left(n_{a}\right)\\ \;0\;else \end{array}\;,$$

where τ_*A*_ is a parameter that sets the time scale of the activation transition. Moreover, the activation of astrocyte “*a*” is signaled back to all its local areas by the following additional rule: The IP_3_ concentration [IP_3_]_*ija*_ of every local area *ij* composing “*a*” is forced to its maximum value ([IP_3_]_*ija*_ = 1) for the entire duration of the active state of “*a*.” Note that, as described by Equation (3), activated astrocytes also release adenosine during the entire duration of the active state.

Finally, transitions from the active to refractory (A → R) and from the refractory to inactive state (R → U) happen spontaneously:

(16) $$P(A\to R)\;=\;\Delta t/\tau_{R},$$

(17) $$P(R\to U)\;=\;\Delta t/\tau_{U}.$$

#### Neuron and Astrocyte Network Spatial Topologies

**Figure 2:**
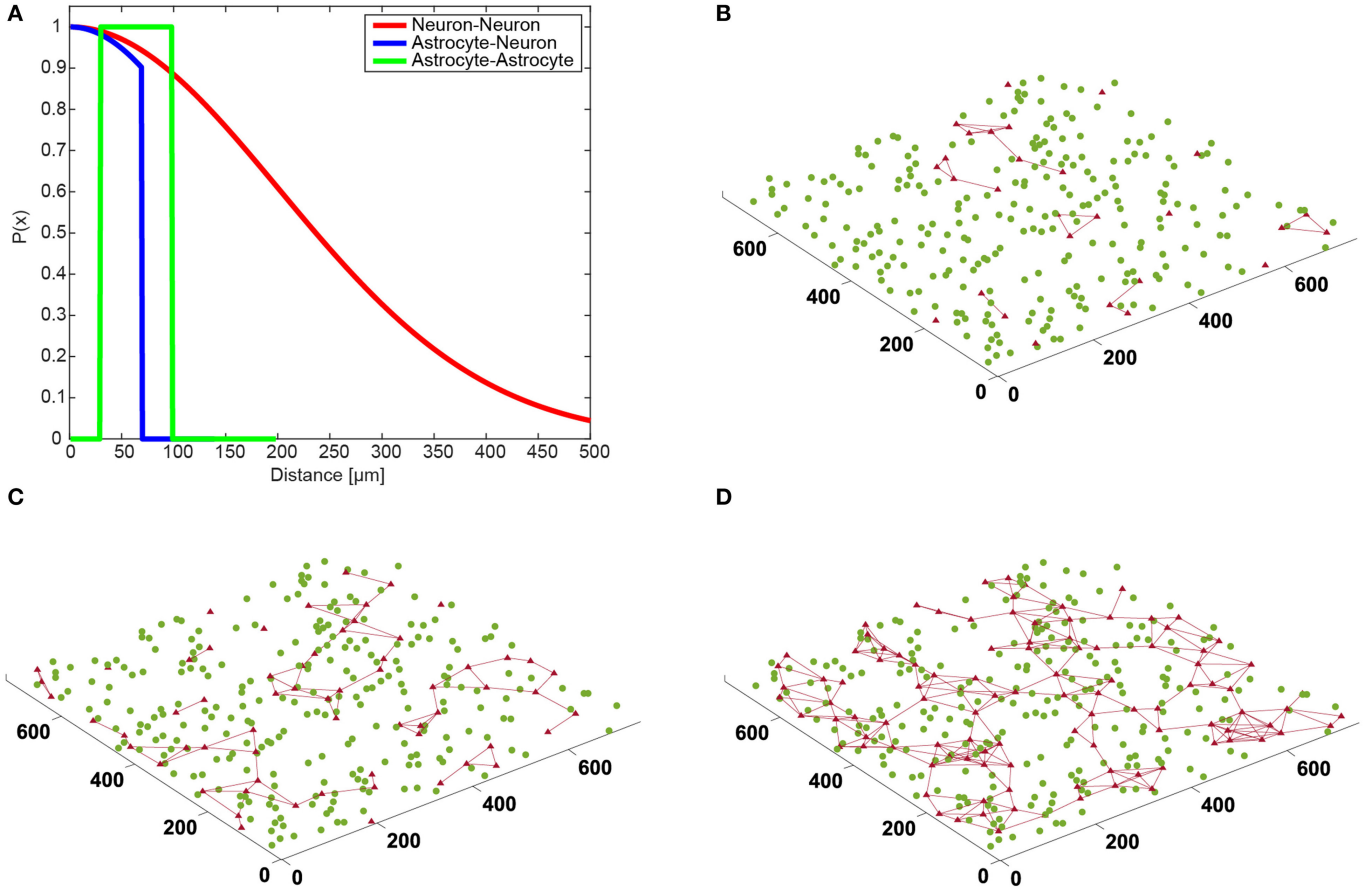
Connection distances between cells and spatial neural network topology. **(A)** Neuron-neuron connections (red) are Gaussian based on distance. Astrocyte-neuron connection probability (blue) follows Gaussian until it reaches a limiter. Astrocyte-astrocyte connections (green) form as long as the two cells are closer than a set limiter. **(B–D)** The graphics show the neuronal network with **(B)** 10%, **(C)** 20%, and **(D)** 30% astrocytes on a “virtual” multielectrode array (units are in μm). The neuronal network is represented by the position of the neurons (green circles), and the astrocytic network includes the cells (red triangles) and the connections between them (red lines).

Astrocytes were randomly placed on a virtual 2D MEA culture surface area of 750 × 750 μm^2^ (with uniform distribution). If the distance between two astrocyte somas was smaller than 30 μm, one of the two astrocytes was randomly relocated until all inter-soma distances were larger than 30 μm. Each astrocyte was connected by gap junctions to every neighboring astrocyte whose inter-soma distance was smaller than 100 μm. Hence, the diameter of one astrocyte is ~100 μm in our model (Figure 2A).

The spatial distribution of the neurons on the virtual MEA was chosen the same way as for astrocytes. However, the method for connecting the neurons differed. Since neurons form long distance connections, we used a connection probability set by a scaled Gaussian distribution:

(18) $$P_{NN}\left(d\right)\;=\;e^{-\frac{d^{2}}{2\sigma_{N}{\;}^{2}}},$$

where *d* is the (inter-soma) distance between two neurons. Each synapse was connected to the nearest astrocyte in a similar probabilistic way, except that a synapse cannot connect to an astrocyte that is farther than a certain cut-off:

(19) $$P_{AN}\left(d\right)\;=\;e^{-\frac{d^{2}}{2\sigma_{A}{\;}^{2}}}\cdot H\left(d_{A}-d\right),$$

where *d* is the distance between the cell body of the nearest astrocyte and the synapse. *H*() denotes the Heaviside function (*H*(*x*) = 1 if *x* > 0, otherwise *H*(*x*) = 0) and *d*_*A*_ is the cutoff distance, which we set to 70 μm (Figure 2A). If the synapse does not connect to the nearest astrocyte, the next-nearest astrocyte is tried and so forth. Note that, in our model, an excitatory synapse can end up without an astrocyte.

### Numerical and Analysis Methods

#### Spike and Burst Detection

**Figure 3:**
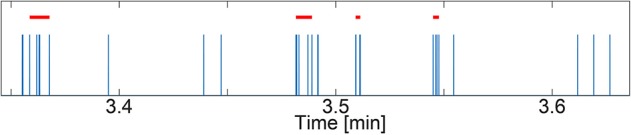
Example spike train (spikes in blue) with detected bursts (red bars) using the cumulative moving average (CMA) algorithm. The simulated spike train stems from a data set with NN+A(30%) and Noise = 0.01. The y-axis shows time in minutes.

In this paper, we analyzed neuronal activity in the form of spikes and bursts which are cascades of spikes. Synchronous population bursts are characteristics of matured and well-connected networks (Giugliano et al., 2004; Wagenaar et al., 2006; Lenk et al., 2016). Spike and burst features were calculated using a modified version of the cumulative moving average (CMA) algorithm (Kapucu et al., 2012; Välkki et al., 2017). The threshold used to decide whether a spike belongs to a burst was set by the skewness of the cumulative moving average of the interspike interval distribution. Using the CMA algorithm, we calculated the spike rate in spikes/minute, the burst rate in bursts/minute, the average burst duration in milliseconds, and the average spikes per burst at the post-synapse. Figure 3 depicts an example spike train from our simulations with detected bursts. For each spike/burst feature and noise level, we performed a one-way ANOVA (GraphPad Prism v8.2.1, GraphPad Software Inc., California, USA) to confirm that the features were statistically different for each model scenario.

#### Frequency and Activity Analysis

We constructed multiple parameter sets describing different neuron or neuron-astrocyte networks. The total spike count of the neuronal network was calculated for each run. The resulting signal was then centered by subtracting its mean, and a discrete Fourier transform (DFT) was applied. We only considered the modulus of the Fourier transform coefficients. For each simulation, we applied the DFT to each of the five conducted runs (see section Simulations) and calculated the corresponding average frequency spectra. The average frequency spectrum was then smoothed by convolution with a Gaussian kernel:

(20) $$\zeta_{s}\left(f\right)\;=\int_{-\infty }^{+\infty }\frac{\zeta \left(x\right)e^{-\frac{{\left(f-x\right)}^{2}}{2\sigma^{2}}}}{\int_{-\infty }^{+\infty }H(y)e^{-\frac{{\left(x-y\right)}^{2}}{2\sigma^{2}}}dy}dx$$

with ζ(*f*) the DFT coefficients and *H*(*y*) = 1 if *y* is between the minimum and maximum frequencies obtained from the DFTs, and 0 otherwise. This allows a correction of border effects. For all frequency spectra shown in this paper, we used σ = 0.025 Hz.

Cross-correlation between neuronal and astrocytic activities was computed by smoothing the neuronal (respectively, astrocytic) activities by

(21) $$L_{s}\left(t\right)\;=\int_{-\infty }^{+\infty }\frac{L\left(\tau \right)e^{-\frac{{\left(t-\tau \right)}^{2}}{2\rho^{2}}}}{\int_{-\infty }^{+\infty }F(y)e^{-\frac{{\left(\tau -y\right)}^{2}}{2\rho^{2}}}dy}d\tau ,$$

with *L* the original pooled neuronal or astrocytic activity signal, and *L*_*s*_ the smoothed signal. *F*(*y*) is equal to 1 if *y* is between 0 and the maximum time of simulation (usually 300 s), and 0 otherwise. We used ρ = 3 s. For each run, we computed the cross-correlation using the crosscorr function in Matlab (version R2017b, MathWorks, USA). The cross-correlation was then averaged across the five runs for each relevant scenario.

Average astrocyte activation ratios were computed for simulations in which astrocytic networks were used. As for neuronal activity, the astrocyte activity was pooled in 5 ms bins; at each time step, the total number of currently active astrocytes in the simulation was recorded. The average astrocyte activation ratios *AR* were then computed by:

(22) $$AR\;=\;\frac{\langle B\rangle }{n_{A}}\frac{{\tau_{R}}_{t}+\tau_{U}+\tau_{A}}{\tau_{R}}$$

with 〈*B*〉 the average number of astrocytes activated at any given time and *n*_*A*_ the total number of astrocytes. $\frac{\langle B\rangle }{n_{A}}$ was thus the average fraction of astrocytes that were activated at any given time. The average transition times between astrocyte states were used to scale the activity such that a value of 1 corresponded to the highest average activity possible (when astrocytes continuously changed from inactivated (τ_*U*_), to activated (τ_*A*_) to refractory (τ_*R*_) states). When applicable, Spearman's rank correlation coefficients and associated *p*-values were computed using the corr function in Matlab.

The homeostatic effects of astrocytes can further be investigated by looking at how the average neuronal spike rate changes when astrocytes activate faster (represented by parameter τ_*A*_), or when the strength of their presynaptic effect is changed (represented by Ω_*g*_). Low values for τ_*A*_ lead to high activation while high values prevent activation [see Equation (15)]. On the other hand, parameter Ω_*g*_ controls the presynaptic effect of astrocyte processes: high values lead to fast recovery of glutamate receptor (and thus low presynaptic effects) while low values lead to slow recovery (and thus high presynaptic potentiation). Therefore, we ran NN+A(30%) simulations with noise *c*_*i*_ = 0.02 and varied τ_*A*_ between 1.0 and 4.5 s and Ω_*g*_ between 0.077 and 51.29 s^−1^.

#### Simulations

To illustrate how the INEXA network model and what the astrocyte contribution to its dynamics is, astrocytic signaling was progressively added, starting from the original INEX model in four sequential stages:

- **Noise only:** we only included the neuronal background noises *c*_i_ (Equation 7), i.e., all synaptic weights and the astrocytic depressing terms were set to zero (*y*_*ij*_ = *y*_Astro_ = 0 in Equation 7). This scenario therefore is to be considered as a reference where the neurons are connected neither to each other nor to the astrocytes.
- **NN only:** we set the synaptic weights to constant values (i.e., −0.7 ≤ *y*_*ij*_ ≤ 0.7), keeping *y*_Astro_ = 0. This stage thus corresponds to a pure neuronal network response with no influence of the astrocytes on the neurons.
- **NN** **+** **PSA:** each excitatory presynapse was connected to an astrocyte (PSA). In this scenario, however, the astrocytes themselves did not form a network (i.e., the term β_*a*_ of Equation 12 was set to zero for all astrocytes at all times) and no adenosine was released into the extracellular space (i.e., we keep *y*_Astro_ = 0 in Equation 7).
- **NN+A(x%):** the complete INEXA model was tested and compared to the second and third phase (i.e., β_*a*_ was computed according to Equation 12 and *y*_Astro_ was set to the value found in Table 1). Furthermore, to test the effect of the number of astrocytes on the network activity, we simulated cultures composed of roughly 10% [called “NN+A(10%)”], 20% [“NN+A(20%)”], and 30% [“NN+A(30%)”] astrocytes.

In all simulations, the network consisted of 250 neurons, of which 200 were excitatory (80%) and 50 inhibitory (20%). Each of the above described simulation phases was run five times with three different noise levels (the upper boundaries of *c*_*i*_ were set to *C*_*max*_ = 0.01, 0.02, or 0.03). The same neuronal network was used in all simulations. However, if present, the astrocytic network was resampled at each run. In total, these four phases produced 18 scenarios. A total simulated time of 5 min was chosen. The values of the parameters used in the simulations are given in Table 1.

#### Topology

Table 2 summarizes the statistics of the simulated neuronal and astrocyte networks. The connectivity within the neuronal network was 29%. Each astrocyte was to connected to between 130 and 250 excitatory synapses depending on the ratio of astrocytes in the network [“NN+A(10%),” “NN+A(20%),” and “NN+A(30%),” more astrocytes yielding less synapses per astrocyte, see Table 3]. Likewise, each astrocyte was connected to one to five neighboring astrocytes through gap junctions depending on the astrocyte ratio (more astrocytes yielding more gap junction couplings per astrocyte).

**Table 2:** Statistics of the neuronal network.

**Measure**	**Value**
Maximum amount of neuronal network connections	62,250
Average number of connections to other neurons	72.12
Network connectivity in %	28.96
Average length of connections in micrometer	211.57
Number of bidirectional connections	5,284

**Table 3:** Statistics of the astrocytic network: mean value and standard deviation over the five runs for NN+A(10%), NN+A(20%), and NN+A(30%), respectively.

**Measure**	**NN+A(10%)**	**NN+A(20%)**	**NN+A(30%)**
Connections of an astrocyte to nearby excitatory synapses	252.05 ± 13.16	194.22 ± 6.15	129.68 ± 1.88
Gap junction connections between astrocytes	1.42 ± 0.56	2.55 ± 0.27	4.86 ±0.31
Lowest and highest gap junction amount (rounded)	0 ± 0–4 ± 1	0 ± 0–5 ± 1	0 ± 1–9 ± 1
Distance between connected astrocytes in μm	68.65 ± 4.78	70.92 ± 1.35	70.14 ± 0.87
Number of excitatory synapses without an astrocyte (rounded)	7363 ± 368	2185 ± 387	544 ± 201
Percent of “naked” (without astrocyte) excitatory synapses	51.06 ± 2.55	15.15 ± 2.68	3.77 ± 1.40

Figures 2B–D shows the spatial topology of neurons and the astrocytic network resulting from the spatial rules described in section Neuron and Astrocyte Network Spatial Topologies. In the case of “NN+A(10%)” (Figure 2B), only a few astrocytes formed connections, and half of the excitatory synapses (51.1%) were not controlled by an astrocyte. In “NN+A(20%)” (Figure 2C), almost all astrocytes were connected to at least one neighboring astrocyte. However, the number of astrocytes used was not enough to reach all synapses, and 15.2% of the excitatory synapses were left without any astrocyte. Finally in “NN+A(30%)” (Figure 2D), a widely interconnected astrocytic network spread all over the entire neuronal network, and only 3.8% of the excitatory synapses were not connected to an astrocyte.

## Results

### Single Synapse-Astrocyte Interaction

**Figure 4:**
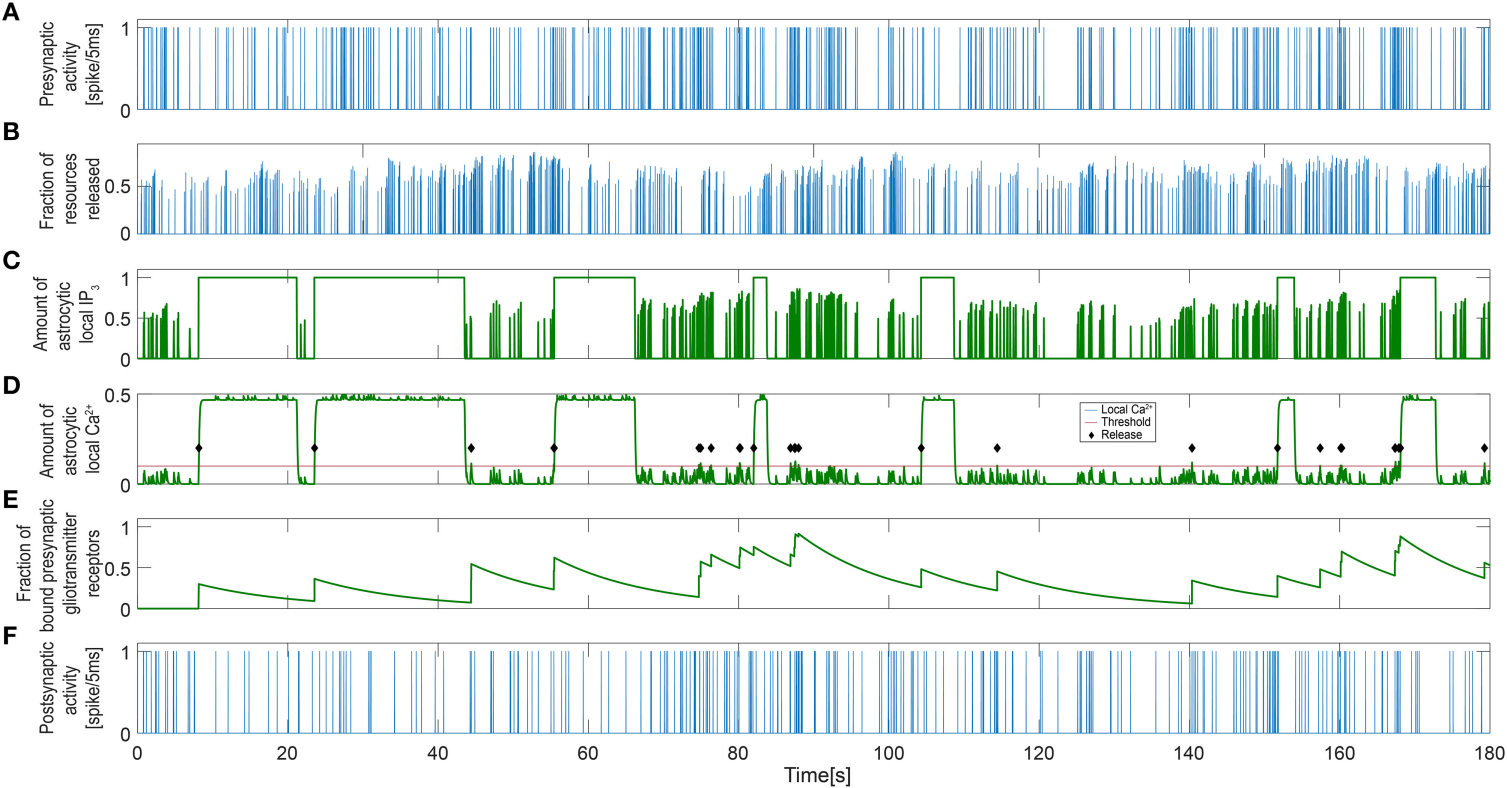
Signaling process governing single tripartite synapse activity. **(A)** Single presynaptic neuronal activity. **(B)** Resources released to the synaptic cleft. The correlation between the release process and the presynaptic activity indicates that this is a spike-induced process. **(C)** Astrocytic local IP_3_ and **(D)** calcium concentration. The levels of local calcium follow those of IP_3_ with a small delay. The different timescales for the neuronal and astrocytic networks are detectable (Equation 9). The red line represents the threshold level for the gliotransmitter release and the green diamonds indicate that gliotransmission has occurred. **(E)** Gliotransmitter glutamate released from the astrocyte controlled by local calcium dynamics. **(F)** Single post-synaptic neuronal activity.

We first use simulation results to illustrate how communication between neurons and astrocytes shapes the dynamics of our INEXA model. Figure 4 shows three time series from a simulation with 30% astrocytes [“NN+A(30%)” scenario]. The release of resources (Figure 4B) was induced by the activity of the presynaptic terminal (Figure 4A), but the amount of neurotransmitters released into the synaptic cleft varied, depending on the fraction of available vesicles (Equation 3) and the fraction of these vesicles that were ready for release (Equation 4). The amount of neurotransmitter in the cleft was directly linked to the post-synaptic activity as described by Equations (5) and (7). Accordingly, more frequent post-synaptic spikes were elicited when larger amounts of neurotransmitters were released (compare Figures 4B,F).

In our model, spike-induced neurotransmitter release had an impact not only on the neuronal network, but also on the astrocytic network. The astrocytes were able to detect synaptic activity through the resources released by the presynaptic terminal in the synaptic cleft. Hence, in response to presynaptic activity, the local astrocyte IP_3_ level increased, which led to the release of calcium from the astrocytic ER (Figures 4C,D). When the astrocyte local calcium concentration exceeded a threshold (the red line in Figure 4D), gliotransmission took place (as indicated by the black diamonds) and a sudden increase in the gliotransmitter concentration was detected (Figure 4E). Gliotransmission signaled back to the synapse, affecting the internal dynamics of the presynaptic terminal: the amount of resources released into the synaptic cleft was therefore higher on average when the gliotransmitter concentration was large (compare Figures 4B,E). Therefore, gliotransmission was release-increasing or potentiating for this particular synapse (see Glial Components). Upon activation of the whole astrocyte, both IP_3_ and calcium levels switched to a high state (Figures 4C,D; the local IP_3_ level is set to 1 upon astrocyte activation). Once activated, the astrocyte released adenosine into the extracellular space, reducing the activity of the post-synaptic neuron, which progressively decreases the spike rate (Figure 4F). In addition, the presynaptic neuron was also indirectly affected by astrocyte activation. The level of local calcium was maintained above the release threshold while the astrocyte was active, which prevented new releases of gliotransmitter. Thus, temporarily canceling the potentiating effect of gliotransmission on the presynaptic terminal [see Equations (5–7)].

**Figure 5:**
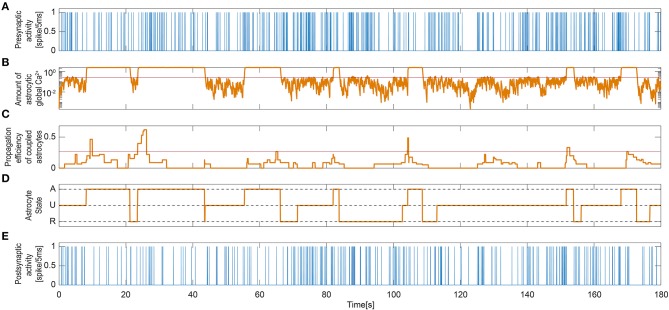
Signaling process governing single astrocyte activity. **(A)** Single presynaptic neuronal activity. **(B)** Astrocytic global calcium dynamics corresponding to the averaged and scaled local responses from all enwrapped synapses. The red line indicates the threshold set for the activation of the astrocyte. **(C)** IP_3_ influx that the current astrocyte receives from all its active neighbors. Again, the red line is the threshold for the activation of the astrocyte. **(D)** State signal of the UAR model astrocyte: inactive dormant state (U), active signaling state (A), and refractory period (R). **(E)** Single Post-synaptic neuronal activity.

As described in the Methods section, the dynamics of astrocyte activation is governed by two variables in our model: the local Ca^2+^ activity from the enwrapped synapses and the contribution to this activity by intercellular Ca^2+^ wave propagation (Equations 12–17). Figure 5 shows the excitation dynamics of the astrocyte connected to the synapse shown in Figure 4. Figures 5A,B demonstrate how the global calcium signal generally increased upon periods of high presynaptic activity. However, the global calcium signal could reach high values even when the presynaptic activity in this particular neuron was weak. This is due to calcium release triggered by other synapses to which the astrocyte was connected. Moreover, the activation propensity of the astrocyte (Figure 5C) depended on the number of its neighboring astrocytes [see Equations (12–13)]. Most of the time, both signals were needed to activate the astrocyte. That means, to activate the astrocyte usually demanded that both the amount of global calcium becomes larger than its threshold and that the activation propensity of the coupled astrocytes crosses over its own threshold. This is for example the case slightly after *t* = 20 in Figure 5, where activation occured when both the calcium trace (panel B) and the propensity trace (panel D) overcame their respective thresholds (red lines). However, having both signals crossing over their thresholds was not mandatory to activate the astrocyte, since astrocyte activation could also be triggered by only one of them. For instance, the activation occurring around *t* = 55 in Figure 5 was triggered when the global astrocyte Ca^2+^ crossed over its threshold, at a time step where the propensity trace was still well below its own threshold.

Figure 5D shows the astrocyte state [inactive (U), active (A), or refractory (R)] along the simulation time. When the astrocyte became activated, the global calcium signal switched to a high state. Those active periods also corresponded to the high state periods observed in the local IP_3_ and calcium signals in Figure 4. The post-synaptic activity was clearly reduced as a consequence of the depression exerted during astrocyte active periods regardless of the activity at the synapse (Figure 5E).

### Spike and Burst Detection

**Figure 6:**
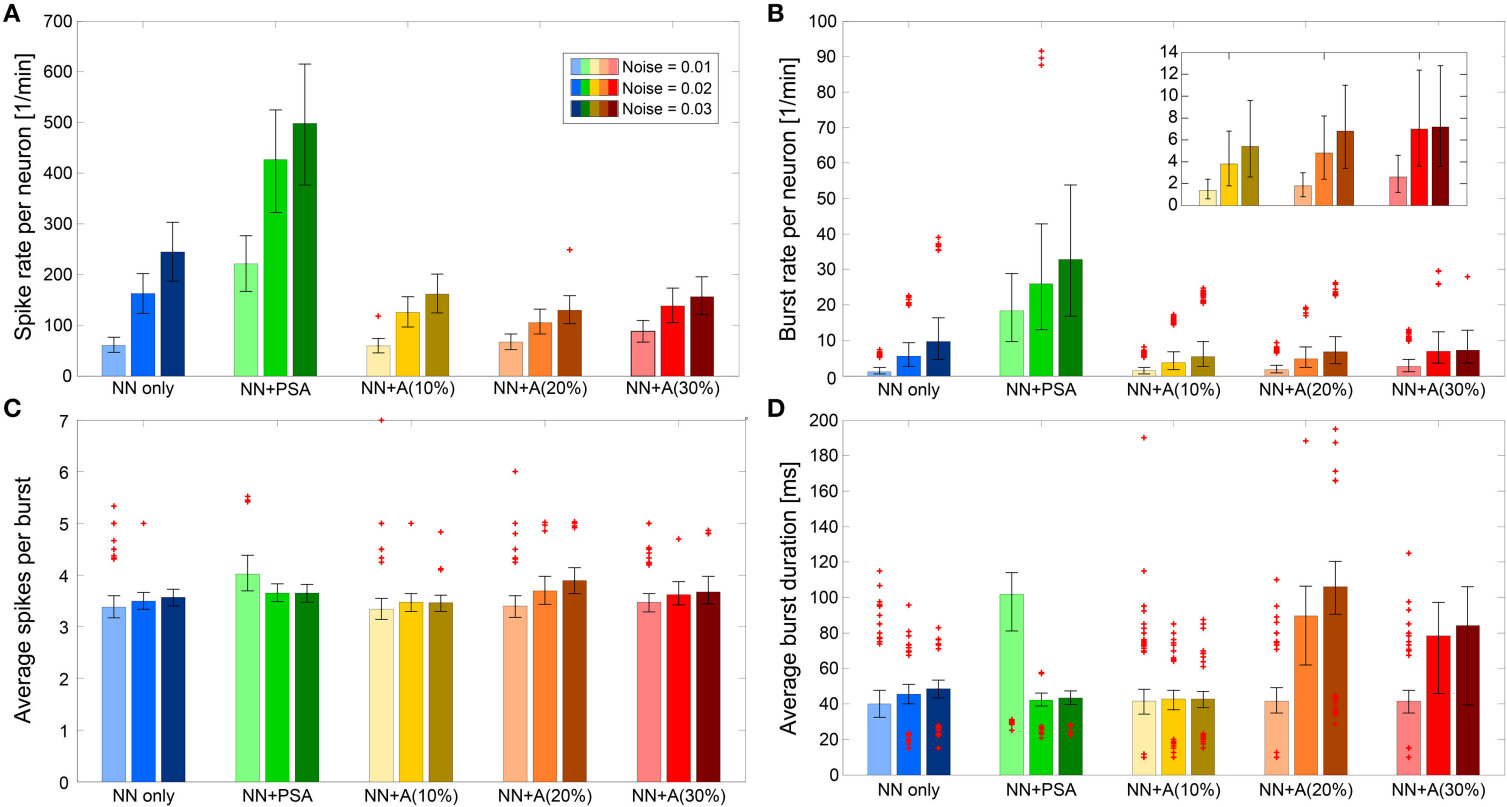
Spiking and bursting behavior features. The features are displayed for the three noise levels in cases of “NN only” (blue), “NN + PSA” (green), “NN + A(x%)” (yellow, orange, red) averaged across five runs, respectively. Error bars plot the 25th and 75th percentiles. Individual plotted points represent extreme data points not considered within the percentiles. **(A)** Average spike rate per neuron per minute. **(B)** Burst rate per neuron per minute. **(C)** Average spikes per burst. **(D)** Average burst duration in milliseconds.

To understand how the local dynamics of the tripartite synapses in the models impacted the dynamics of the whole network, we next quantified the bursting behavior of the neuronal network for each simulation scenario (see section simulations above), especially when presynaptic astrocyte signaling and the formation of astrocytic networks were added to the model. Figure 6 shows the burst and the spike rates as well as the number of spikes per burst and the burst duration in each of the studied simulation scenarios (except for the “noise only” scenario that, as expected, exhibited no remarkable bursting).

When the neuronal network was formed via synaptic connections that did not depend on astrocyte activity (“NN only,” the blue bars in Figure 6), the spike rate increased with the noise level, since the noise level determined basal firing activity. Those spikes proportionally contributed to the burst development as indicated also by the higher burst rate. However, Figures 6C,D shows that the characteristics of the bursts (number of spikes per burst, burst duration) were not affected by noise level.

In the “NN+PSA” case, where the astrocytes were connected to the presynaptic terminals of the neuronal network but not to each other (the green bars in Figure 6), the network as a whole became more active as a result of the potentiating effect of the astrocytes on the excitatory synapses. As one might expect, the spike rate increased with the noise level/basal rate (Figure 6A). Moreover, the burst duration decreased since the number of spikes per burst was constant, but the burst rate increased. These changes were the consequences of the gliotransmitters released from the astrocytes. On average, gliotransmission increased the presynaptic release probability [see Equation (7)], which led to a larger amount of resources released into the synaptic cleft [see Equation (5)], and thus a larger firing rate of the post-synaptic neuron compared to the “NN only” scenario.

The addition of the astrocytic network to the model strongly changed the bursting behavior of the neuronal network. In those “NN+A(x%)” scenarios, we both introduced astrocyte to astrocyte coupling via gap junction, but also the depressing impact of astrocytes on the post-synaptic firing rate. The immediate effect of the addition of the astrocytic network was that both the spike rate and the burst rate were much lower than those obtained in the “NN+PSA” case (Figures 6A,B) while the mean number of spikes per burst was not altered (Figure 6C). Interestingly, the spike rate was almost constant regardless of the number of astrocytes [compare the different “NN+A(x%)” scenarios] because of the trade-off between the effect of glutamate transmission and adenosine depression. However, as can be seen in the inset of Figure 6B, the burst rate slightly increased with the number of astrocytes, which suggested that one of the consequences of the astrocytic network might be the introduction of bursting behavior.

Analyzing the effects on burst duration was more complex. In the case of “NN+A(10%)” (the yellow bars in Figure 6), the average burst duration did not significantly change with the introduced noise levels. However, the high number of outliers for the average burst duration revealed the existence of two types of behaviors within the neural network for intermediate-to-high noise levels (Figure 6D). This might result from an astrocytic network that was too sparse to compensate for the high activity of the neural network with high noise. Indeed in “NN+A(20%)” and “NN+A(30%),” the burst duration increased with increasing noise and with respect to “NN+A(10%).” These results support our above interpretation: as the number of astrocytes increased, the astrocytic network was also strengthened. Thus, it was able to control the whole neuronal network by preventing it from overexcitation, even at high noise levels.

One-way ANOVA confirmed that the spike and burst features were significantly different for each model scenario (*p* < 0.0001). We performed the test for each feature and noise level separately. Taken together, Figure 6 shows that the astrocyte network downregulated the activity of the neural network by decreasing its burst and spike rates while increasing burst duration.

### Activity and Frequency Analysis

To further analyze how the addition of presynaptic astrocyte signaling and full astrocytic networks affected neuronal activity, we next quantified the changes in the overall activity levels and in specific frequency bands of the neuronal network activity. Therefore, we applied discrete Fourier transforms (DFT) on the pooled neuronal activity signals (details in the Methods section).

#### Effect of Presynapse-Astrocyte Processes

**Figure 7:**
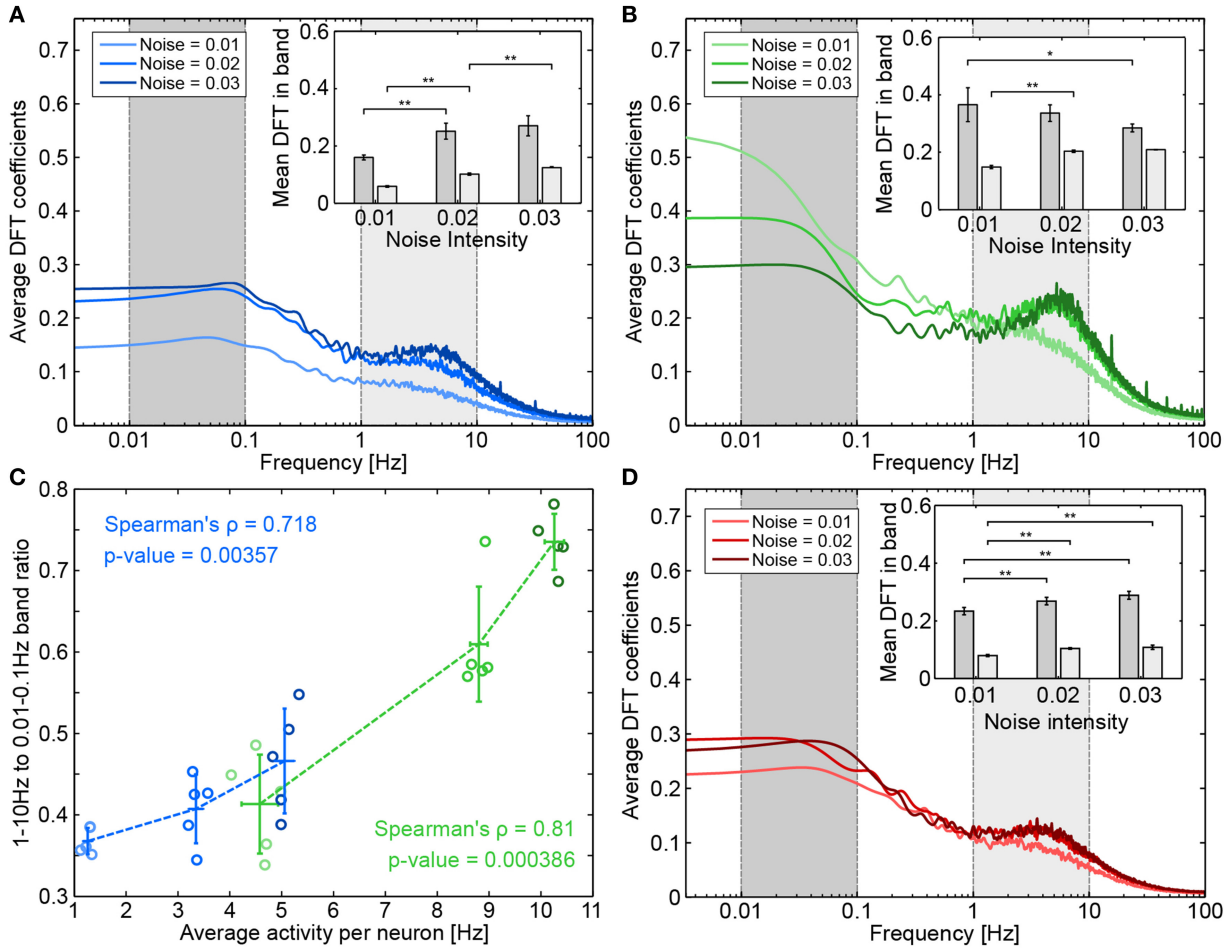
Effect of presynapse-astrocyte processes on neuronal activity. Raw frequency spectrums for **(A)** “NN-only” **(B)** “NN + PSA” and **(D)** “NN+A(30%)” were averaged across five runs and smoothed as described in the Methods section. The inset shows the average DFT coefficients for different noise intensities and for two frequency bands: 0.01–0.1 Hz (dark gray) and 1–10 Hz (light gray). Error bars plot the standard deviation of band averages across runs. Significance was assessed by double-sided Mann-Whitney tests (comparing distributions of band averages). *p < 0.05, **p < 0.01. **(C)** Relationship between average activity per neuron and the ratio between band averages. Each circle represents a run, a darker circle denotes a higher noise intensity; blue data corresponds to “NN only” and green data corresponds to “NN + PSA”.

The “NN only” scenario is a natural comparison point for understanding the effect of astrocytes on neuronal activity. Figure 7A shows the frequency spectra corresponding to the “NN only” scenario for different levels of noise. The frequency spectra display a slight increase for two frequency decades: very low frequencies, between 0.01 and 0.1 Hz (the dark gray band); and medium frequencies between 1 and 10 Hz (the light gray band). As noise intensity increased (the light to dark blue curves), the amplitude of both frequency bands increased. However, as can be seen in the inset of Figure 7A, in which both frequency bands were averaged, the gap between them seemed to decrease as the noise intensity increased.

When presynaptic astrocytes were added (“NN+PSA”), the average intensity of both bands strongly increased (see the green bars in Figure 6A). Gliotransmitter release from the astrocyte increased the value of the basal release probability $U_{ij}^{*}$ of TM synapses (De Pittà et al., 2011), which thus increased the amount of released resources. The corresponding frequency spectrums can be seen on Figure 7B. While the power in the 1–10 Hz band seemed to increase with noise intensity, the power in the 0.01–0.1 Hz band actually decreased. The increase of the average neuronal activity evidenced by Figure 6 is thus not uniformly distributed across frequencies.

Since noise intensity was linked to increased average activity, we checked whether the changes in medium and low frequency bands could be linked to average activity in both the “NN only” and “NN+PSA” scenarios. We thus examined how the ratio between the 1–10 Hz and the 0.01–0.1 Hz bands changed as a function of average activity. Figure 7C shows these values for both “NN only” (blue) and presynaptic astrocyte signaling (“NN+PSA,” green) scenarios. In both cases, increases in average activity were significantly correlated with increased band amplitude ratios, meaning that increased spiking activity mostly influenced the higher medium frequencies as opposed to low frequencies. This agreed with the spike and bursts analysis since in the “NN only” and “NN+PSA” scenarios, the increase in the burst rate per neuron with the noise seen in Figure 6 could be associated with the increase in the amplitude of the 1–10 Hz band.

#### Effect of Astrocytic Networks

The addition of a full astrocytic network—which could potentially synchronize distant synapses and depress the whole neuronal network through adenosine release—changed how the neuronal network behaved. With respect to “NN only” simulations (the blue bars in Figure 6A), the average activity of the neural network (the yellow to dark red bars) was slightly increased by the astrocyte network for low noise intensity (the left-most bars of each group), but it was strongly decreased for high noise intensities.

**Figure 8:**
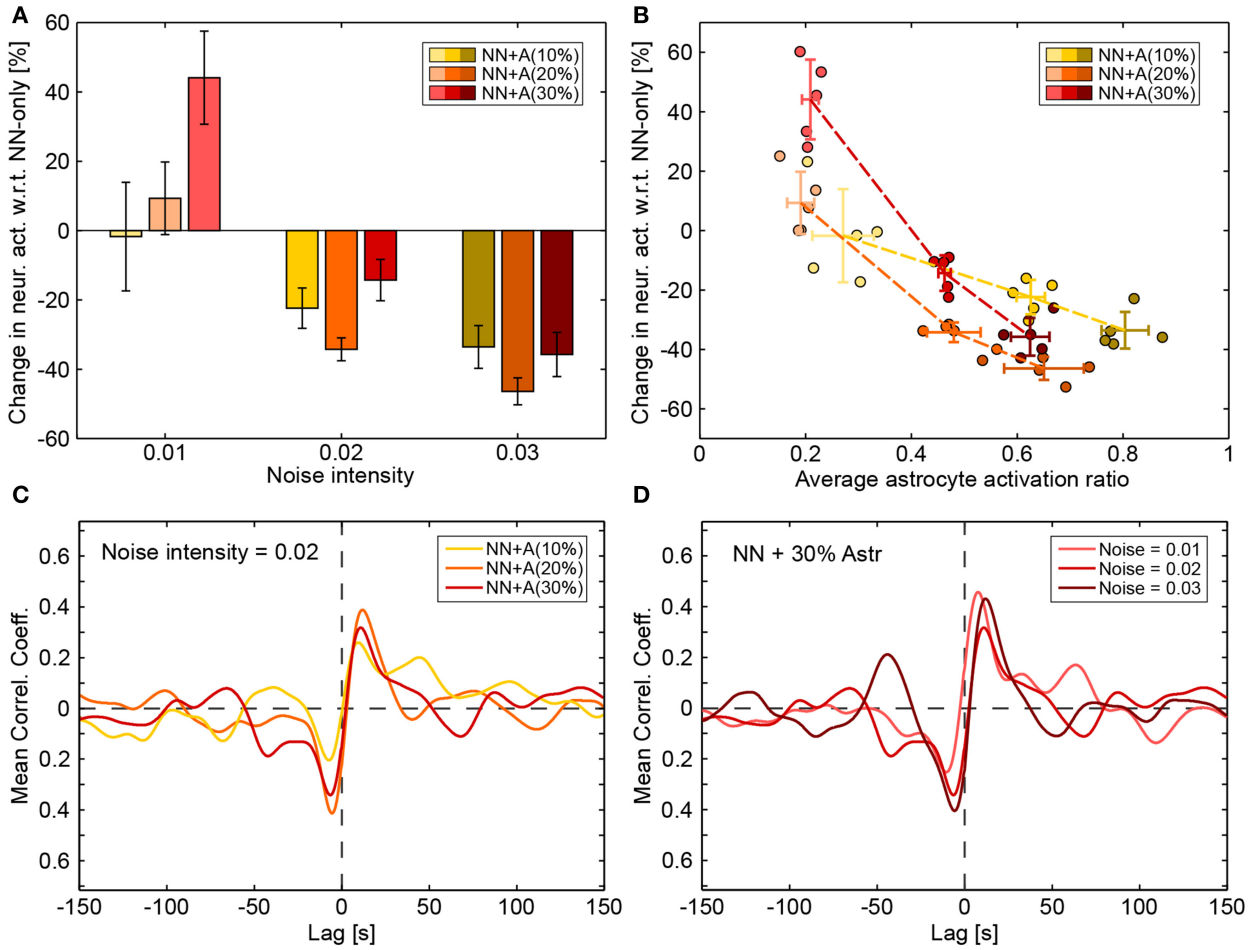
Effect of astrocytic networks. **(A)** Changes in neuronal activity introduced by the addition of astrocytes (compared with “NN only” simulations). Values were averaged across runs and error bars plot the standard deviation across runs. **(B)** Changes in neuronal activity introduced by the addition of astrocytes (compared with “NN only” simulations) as a function of the average astrocyte activation ratio (a value of 1 denotes the highest possible activity in the astrocytic network). Each circle represents a run. Darker circles denote higher noise intensity and the hue (yellow to red) denotes the amount of astrocytes in the simulation (10–30%). Crossed error bars indicate averages and the standard deviation across the runs. **(C)** Average cross correlations between neuronal and astrocytic smoothed activities for a constant noise intensity of 0.02 and for “NN+A(10%)” (yellow), “NN+A(20%)” (orange), and “NN+A(30%)” (red). **(D)** Cross correlation between neuronal and astrocytic smoothed activities for varying noise intensity (light to dark red) in the “NN+A(30%)” scenario. Cross correlation values were computed as described in the Methods section.

Figure 7D shows the average frequency spectra obtained when 30% of astrocytes were present (corresponding figures for 10 and 20% show similar results). In contrast to the above results, when the noise intensity increased, the frequency spectrums did not change greatly and stayed close to the frequency spectrums of “NN only” simulations (Figure 8A). As the average band intensity increased with the noise intensity, as shown in the inset, the strength of both low (dark gray) and medium (light gray) frequency bands slightly increased as well. In contrast to the “NN+PSA” scenario, the 1–10 Hz frequency band did not increase much with increasing noise. Figure 8A shows how astrocytic networks affected neuronal activity by displaying the change (in %) between “NN only” and “NN+Astr(x%)” simulations (yellow to red corresponds to 10 to 30% astrocytes) for increasing noise intensities. Increasing the number of astrocytes in the networks had two opposing effects: (1) It introduced more enwrapped synapses, which, as already mentioned, increased the average neuronal activity. (2) It decreased neuronal activity by releasing ATP/adenosine upon astrocyte activation. In case astrocytes were not stimulated enough to be consistently activated, adenosine release was rare and effect (2) was weak compared to (1). With low noise, the low average neuronal activity therefore explains the increase of activity seen in Figure 8A, because effect (1) was greater than (2). On the other hand, when the noise increased (noises 0.02 and 0.03), adenosine signaling was more frequently activated, and the overall effect of the astrocyte network was to decrease activity when compared to the “NN only” scenario.

The interplay between astrocyte activation and changes in neuronal activity can clearly be seen in Figure 8B: high astrocyte activity clearly correlated with decreased neuronal activity while low astrocyte activity correlated with increased neuronal activity. The higher the number of astrocytes, the steeper this relationship became (yellow to red curves). With enough astrocytes, the interplay between neuronal and astrocytic networks even impacted the cross-correlation between average neuronal activity and average astrocyte activity. Figure 8C shows the average cross-correlation between neuronal and astrocytic activities for increasing number of astrocytes (yellow to red) at a constant noise intensity. Figure 8D shows the same cross-correlation but only for the “NN+A(30%)” scenario and for increasing noise intensities (light to dark red). In all cases, neuronal and astrocytic activities were negatively correlated with lags around −5 s (global minimum of the mean correlation coefficient) and positively correlated with lags around 10 s (global maximum of the mean correlation coefficient). This means that high astrocyte activity was followed by low neuronal activity ~5 s later, while high neuronal activity was followed by high astrocyte activity ~10 s later (which is of the order of the time needed by an astrocyte to activate).

**Figure 9:**
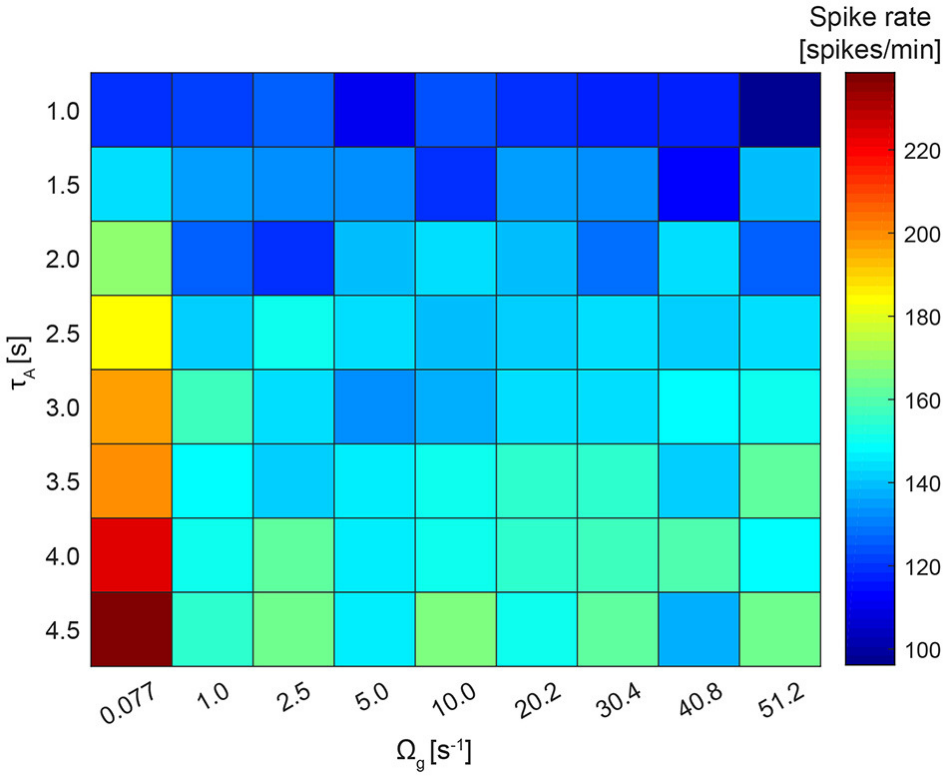
Network firing stability. The recovery rate of the gliotransmitters, Ω_*g*_, varies between 0.077 and 51.2 s^−1^ and the average activation time of an astrocyte, τ_*A*_, between 1.0 and 4.5 s. For this simulation, the NN+A(30%) model and *c*_*i*_ was fixed to 0.02 was used. For each run, the average across the resulting spike rates of all 250 neurons was calculated.

To explore if astrocytes contribute to network firing stability as a homeostatic modulator, we varied the recovery rate of the gliotransmitters, Ω_*g*_, and the average activation time of an astrocyte, τ_*A*_ (Figure 9). As expected, increasing τ_*A*_ led to a decreased neuronal activity across the whole range of Ω_*g*_ values. Increasing Ω_*g*_ resulted in a decreased presynaptic potentiation, and thus in a decreased average spike rate. No further changes could be seen for Ω_*g*_ > 1 s^−1^, since presynaptic glutamate receptors recover very fast and prevent any presynaptic potentiation. The resulting average spike rate thus resulted from a trade-off between local astrocyte processes (whose potentiating effect is controlled by Ω_*g*_) and global astrocyte activations (whose depressing effect is controlled by τ_*A*_).

To summarize, our simulations revealed that astrocytes exerted two opposite effects on neuronal activity. The activation of presynaptic astrocyte processes increased the neuronal activity through the release of potentiating gliotransmitters like glutamate. When neuronal activity became high enough to elicit significant astrocyte activation, depressing gliotransmitters like ATP/adenosine were released, leading to a decrease of the neuronal activity. Overall, these results show that astrocytic networks promoted stabilization of the average neuronal activity, boosting low average neuronal activity through the effect of presynaptic astrocyte processes while reducing high activity levels through adenosine release.

## Discussion

We developed an *in silico* description of connected neuronal and astrocytic networks and assessed their interactions combining in a biologically plausible fashion previously introduced models for different parts of those networks (De Pittà et al., 2011; Lenk, 2011; Lallouette et al., 2014). Our goal was to study the role of astrocyte networks when coupled to neuronal networks. To assess the effects of the astrocyte networks on the neuron network, we quantified spike and burst features and used pooled spike trains as indicators of frequency based activity at the network level. The frequency analysis of the pooled spike trains allowed us to identify changes in the signaling patterns of the network.

Astrocytes may play a role on short-term and long-term synaptic plasticity (De Pittà et al., 2016). Short-term plasticity includes the potentiation or depression of neurotransmitter release, which occurs in the milliseconds to minutes range. Astrocytes were also connected to influence long-term potentiation or depression (Turrigiano, 2008; De Pittà et al., 2016). Memory and learning related changes of the global synaptic strengths could be a result of adjustments to an increasing or decreasing firing rate. However, they could also be related to more local homeostatic effects (Turrigiano, 2008).

With our model, we have mainly investigated short-term effects. Comparing the spike and burst features between the pure neuronal network (“NN only”) and the neuronal network where each excitatory presynapse was connected to an astrocyte (“NN+PSA”), our simulations show that more noise means more activity, because of the absence of depression mechanisms stronger than the short-term depression introduced by the Tsodyks-Markram synapses. When astrocytes are introduced to the model, we can observe two types of responses from the network as compared to “NN only.” On the one hand, when the average activity is low (noise = 0.01), the astrocytes promote neuronal activity, since the presynaptic effect of the astrocytes prevails over adenosine depression. On the other hand, when the average activity is higher (noise = 0.02 and 0.03), neuronal activity decreases due to astrocyte effects, meaning that the depression effect prevails over the presynaptic signaling. Additionally, the longest bursts are obtained in simulations where the astrocytes form a significantly coupled network [especially “NN+A(20%)” and “NN+A(30%)”].

Our results therefore suggest that astrocytes may stabilize the activity of the neuronal network on a short-term (De Pittà et al., 2016): the astrocyte network would decrease neuronal activity through adenosine release when it is high or increase it through release-increasing presynaptic signaling when it is low. This homeostatic mechanism is based on the competition between two short-term synaptic plasticities regulated by gliotransmission: (1) gliotransmitter-based short-term increase of glutamate release by the presynaptic element and (2) short-term depression of the synapse via depressing gliotransmitters like adenosine. The system is homeostatic because (1) dominates (2) when neuronal activity is low, whereas (2) dominates (1) when neuronal activity is very large. That astrocytes could act as homeostatic regulators of the neuronal network activity has already been suggested based on the experimental observation that astrocytes release TNFα in response to prolonged periods of neuronal inactivity (De Pittà et al., 2016). At long time scales (hours to days) the released TNFα is expected to strengthen excitatory synapses while depressing inhibitory ones, thus contributing to the restoration of activity in the neuronal network (De Pittà et al., 2016). Our model adds to this possibility suggesting that astrocytes could also bring forth a further homeostatic mechanism based on competing processes of synaptic plasticity that could occur on fast time scales of the order of second or minutes. Consequently, future studies are required to better understand how astrocyte-mediated homeostasis on different time scales could ultimately mold neuronal network activity.

To investigate further if astrocytes contribute to network firing stability, we altered the recovery rate of the gliotransmitters and the average activation time of an astrocyte in case of “NN+A(30%).” As expected, the firing rate increased when the astrocytic activation time was increasing. Thus, the inhibiting effect of astrocytes—that dominates over the potentiating one—was diminished. For a longer recovery rate of the gliotransmitters, the astrocytes did not seem to have a clear effect on the network firing. The reason might be that the recovery/degradation was much faster than the time scale of neuronal activity.

Savtchenko and Rusakov (2014) presented a ring-like network model including pyramidal neurons and fast-spiking interneurons as well as volume-limited regulation of the synaptic efficacy. They used this latter mechanism as a way to emulate the spatially constrained effects of gliotransmission. The depression, e.g., upon astrocytic adenosine release, of the excitatory signals to the interneurons resulted in a decreased firing rate and network synchronization. In contrast, the facilitation. e.g., upon glutamate release, increased the firing rate while not altering much the network synchronization. In our simulations, the synaptic regulation from each astrocyte was also volume-limited but the astrocytes were inter-connected, allowing sequential activation of neighboring astrocytes. In addition, Savtchenko and Rusakov (2014) decoupled the potentiation or depression of synapses from the actual neuronal activity. In contrast, our simulations implemented a feedback loop between neuronal and astrocytic activity. Taken together, these differences make it unclear whether the same effects on network synchronization could be observed once the feedback loop is closed.

Recently, Paavilainen et al. (2018) compared hiPSC co-cultures aged 8+ weeks with hiPSC co-cultures aged 15+ weeks containing neuron and astrocyte networks. They observed a slight decrease in the spike rate for the hiPSC co-cultures aged 15+ weeks, together with an increase of the burst rate and duration, while the number of spikes per bursts was constant. Importantly, the hiPSC co-cultures aged 8+ weeks contained about 5% astrocytes and the hiPSC co-cultures aged 15+ weeks contained about 25 % astrocytes. Comparing our simulation results with 30% astrocytes to those with 10% astrocytes produces similar results (increased burst rate and duration, no change in spike count per burst), although the spike rates are similar in our case. Therefore, our model predicts that the change in activity observed in Paavilainen et al. (2018) could be due to the change in the astrocyte/neuron ratio. Currently, our computational model is established in 2D to resemble experimental *in vitro* data. However, it can be easily extended to 3D, and thus can give more insights on *in vivo* data.

While all of the mechanisms, pathways, and released gliotransmitters described in this paper have been adapted from astrocyte studies, the biological evidence that they co-exist in a single astrocyte is still sparse (Covelo and Araque, 2018). It is thus possible that the effects are a result of separate astrocyte populations or even astrocytes in different brain regions, just as neurons differ from one area to another. However, our model can simulate many of the subsets of astrocytic and neuronal mechanisms. Predictions about the functional role of astrocytes in neural networks are conceivable. In the future, it will be possible to adjust the model to specific combinations or even brain areas with differently functioning neurons and astrocytes.

To conclude, we have developed a neural network model in order to study the effect of astrocytes on neuronal network behavior. Our simulations show that astrocyte networks can act as homeostatic controllers with release-increasing and depressing effects on the synapse. These effects act on two different time scales for astrocytes and neurons. Our simulations suggest that tripartite synapses alone are not enough to produce these effects, and thus, the astrocytic network dynamics based on IP_3_-controlled calcium waves are essential for understanding how astrocytes modify neuronal communication. The model presented here provides a basis for further studies of neural interaction and the relevance of this interaction for brain function.

## Data Availability Statement

Both the code and the raw data supporting the conclusions of this article will be made available by the authors, without undue reservation, to any qualified researcher.

## Author Contributions

KL, ES, AL-G, and JH designed and performed research. ES, KL, AL-G, and JL wrote analysis tools and analyzed the data. ES and KL wrote the first draft of the manuscript. KL, ES, JL, AL-G, HB, and JH contributed to the manuscript writing and revision. In addition, they have read and approved the submitted version.

### Conflict of Interest

The authors declare that the research was conducted in the absence of any commercial or financial relationships that could be construed as a potential conflict of interest.
